# Use Heavy Foils?

**Published:** 1870-04

**Authors:** 


### Editorial.

### "USE HEAVY FOILS?"

YES, in almost every filling, varying, however, in each case, in the proportion of heavy foil with the light, Nos. 4 or 6.

With our present experience, we prefer Nos. 30, 60 and 120.

In almost every filling, we use No. 4 for the foundation and indeed, for filling up from two-thirds to three-fourths of all deep cavities, then filling the remainder, and finishing with heavy foil. In ordinary cavities the whole filling may be made with heavy foil, No. 30 or 60, the former is preferable for the beginning. The form, size and location of a cavity to be filled should always determine the precise form of the material to be used.

In the use of heavy foil, the aim should always be to place it upon the receiving surface, a smooth and free from wrinkles or crimping as possible, and so condense it. In large cavities, the foil should be cut, so that it would lay with a plain surface when introduced, except, that perhaps, it may be well to have the pieces slightly larger than the cavity in order that they may curve up a little against the wall. Heavy foil is far more easily condensed than light, and the receiving surface more easily retained.

When a filling is brought up well nigh to the orifice of the cavity, heavier foil may be used, and the pieces may be made larger than the diameter of the orifice, so that when it is condensed all over the surface, there will be a portion to fold over, and consolidate against the border. By this method of manipulation, a far more solid and perfect margin can be made to the filling, than is usually made with thin foil, and the adaptation to the border of the cavity can also be made more perfect.

This method, also, renders it an easy matter to bring up the margins of a filling more rapidly than the central portion which is oftentimes desirable in medium and large cavities on the masticating surfaces of the molars.

The formation of contour fillings is much easier with heavy than with light foil, and the welding is far more perfect with proper manipulation.

We have heard the remark made several times, mostly by those who have not used it, that "there will be a large amount of very poor work done with heavy foil;" to this we always assent, and to this we can usually add, that there is a large amount of very inferior work done with Nos. 4 and 6, and with every form of gold that is used. We see very inferior operations with light foils, almost daily, but we have not thought of that being a reason why we, or anybody else should abandon their use, but we do see, in such fillings, an unanswerable argument for such operators learning to do better, or cease their miserable botchery.

There are certain modes of operating in which heavy foils will be totally impracticable. Those who practice wholly by these modes should confine themselves to light foils.

There is, too, a class of persons who require years of training to develop anything like a good degree of manipulative ability. Such persons do not readily take up and conform themselves to new modes and processes; all such persons, if they attempt any change, should go very slowly, and under the influence of the most favorable circumstances, otherwise in their hands, every new thing will be a failure.

There is another class of persons who, with great avidity—without discrimination, consideration, or the exercise of judgment, seize upon every new thing that is presented—mount every new hobby that comes along, and ride it with whip and spur, till they are thrown off and incontinently whirled from enthusiasm into disgust.

Now we have no objection to the hobby rider, but we do object to the man, who says there is no other horse in the world but the one he is riding. We know a few of this sort who after they were dismounted denied that they were riding, and further, that there wasn't any horse to ride. We would advise such not to go into heavy foil speculation.

It is best for everyone who *knows* he can't swim, to stay where he can keep his feet on bottom. T.

